# Psychological and behavioral processes in stroke survivors with recovery exceeding prognostic expectations: a qualitative study of positive deviance

**DOI:** 10.3389/fresc.2026.1743882

**Published:** 2026-06-29

**Authors:** Ryu Kobayashi, Norikazu Kobayashi

**Affiliations:** 1 Department of Occupational Therapy, International University of Health and Welfare, Narita, Chiba, Japan; 2 Department of Occupational Therapy, Tokyo Metropolitan University, Arakawa, Tokyo, Japan

**Keywords:** activities of daily living, occupational therapist, positive deviance, prognosis, reflexive thematic analysis, stroke

## Abstract

**Background:**

Some patients demonstrate exceptional post-stroke recovery beyond prognostic expectations; this phenomenon is referred to as positive deviance. Understanding how such recovery is perceived in clinical practice may provide insights for improving rehabilitation.

**Aim:**

This study aimed to explore how occupational therapists perceive and interpret psychological and behavioral processes observed among stroke survivors whose activities of daily living (ADL) improvement exceeded prognostic predictions.

**Methods:**

A qualitative descriptive design was employed. Semi-structured interviews were conducted with eight occupational therapists working in a rehabilitation ward. The analysis focused on psychological and behavioral processes in patients with post-stroke ADL improvement beyond prognostic expectations, as perceived by the therapists. Data were analyzed inductively using reflexive thematic analysis.

**Results:**

Six themes were identified as common patterns of psychological and behavioral processes among the patients: 1) maintaining own identity based on occupation; 2) positive and flexible problem-solving orientation; 3) mental fortitude to face challenges; 4) self-management toward goal attainment; 5) development of self-efficacy through accumulated successful experiences; and 6) proactive use of social support.

**Conclusions:**

The psychological and behavioral processes associated with positive deviance, as perceived by occupational therapists, provide a clinically grounded perspective on stroke recovery and may inform the development of rehabilitation strategies to enhance recovery outcomes.

## 1 Introduction

To maximize the efficiency and effectiveness of rehabilitation in stroke survivors, it is crucial to accurately predict outcomes starting from the early stages of hospitalization. In particular, activities of daily living (ADL), such as dressing, eating, and toileting, are closely linked to discharge destination, social participation, and quality of life (1–3). ADL prognosis is of great interest not only to healthcare professionals but also to patients and their families, as accurate prediction of ADL outcomes plays a vital role in setting achievable goals for patients, facilitating interprofessional information sharing, determining the appropriate length of stay, guiding patient and family decision-making, and making discharge arrangements (4, 5).

Various factors, including age, baseline neurological status, upper limb paralysis, and initial ADL performance, are associated with ADL outcomes in stroke survivors (4, 6). Using these factors, several prediction models have been developed to estimate ADL scores at discharge with relatively high accuracy (7–9). However, even when applying these models, a certain proportion of patients show ADL improvements that far exceed predictions. Kobayashi et al. evaluated the performance of a predictive model for the motor Functional Independence Measure (motor FIM) score in 207 patients who were undergoing post-stroke rehabilitation; although the model showed high predictive accuracy, approximately 25% of the individuals exceeded the predicted scores by more than 10 points (10). Furthermore, these individuals could not be readily identified based on known factors, such as age, sex, stroke type, or FIM scores at admission (10).

Traditionally, such exceptional individuals were considered statistical outliers and thereby frequently excluded from analyses to improve the accuracy of prognostic prediction models. However, in recent years, outliers who demonstrate markedly better outcomes than predicted have been conceptualized within the framework of positive deviance (PD)—a perspective increasingly recognized as valuable for addressing complex challenges in healthcare (11). Patients who demonstrated this tendency may exhibit distinct patterns of psychological and behavioral processes that are not captured by conventional prognostic models. Identifying such factors and processes could provide important insights for improving the quality and personalization of rehabilitation practice. Indeed, previous studies have highlighted the role of factors, such as motivation and self-efficacy, in facilitating ADL improvements (12), which suggests that modifiable factors or processes may contribute to the unexpectedly positive recovery trajectories that are observed in patients who demonstrated PD.

The Model of Human Occupation (MOHO) (13), a leading conceptual framework in occupational therapy, provided sensitizing concepts that informed the clinical framing of this study—not as a deductive analytical framework, but as a conceptual background for situating the research questions within occupational therapy theory. The MOHO conceptualizes human occupation as emerging from the dynamic interaction of volition—encompassing values, interests, and personal causation—habituation, performance capacity, and the environment. From this perspective, recovery is shaped not only by neurological and physical factors but also by a patient's sense of occupational identity, motivation for engagement, and ability to mobilize internal and external resources. Existing quantitative prognostic models for stroke rehabilitation effectively capture biomedical predictors but do not account for these psychological and behavioral dimensions. This constitutes a theoretical gap: it remains unclear whether patients who demonstrate PD exhibit distinct patterns of psychological and behavioral processes that facilitate recovery beyond prognostic expectations, and if so, what those processes look like in clinical practice.

The patterns of psychological and behavioral processes associated with PD in patients who demonstrated exceptional recovery are likely to be recognized through tacit knowledge that has been accumulated by healthcare professionals who observe and support patients’ recovery processes over extended periods in clinical settings. Among rehabilitation professionals, occupational therapists play a particularly vital role, as they specialize in supporting individual engagement in activities, including ADL, while attending to each patient's individual patterns of engagement and response within the therapeutic process. This close and sustained engagement positions occupational therapists to observe not only patients’ physical functioning but also the psychological and behavioral dimensions of recovery—such as motivation, self-directed effort, and responses to challenge—that resonate with the volitional and occupational constructs articulated in the MOHO. Therefore, clinical insights based on the experiences of occupational therapists may offer important clues for identifying factors that underlie PD that are not captured by conventional prognostic models.

Importantly, these factors should not be conceptualized as static attributes fixed within the individual. Contemporary rehabilitation science increasingly recognizes that motivation and engagement in rehabilitation are dynamic, co-constructed processes shaped by the therapeutic relationship and clinical interactions (14). A growing body of literature on therapeutic alliance—defined as the collaborative bond between therapist and patient—highlights its significant contribution to rehabilitation outcomes (15). From this perspective, factors associated with PD may emerge and be cultivated through skilled therapeutic interaction, rather than being predetermined by patient-level characteristics alone. This framing has direct implications for occupational therapy practice, suggesting that therapists may play an active role in fostering the very processes that underlie exceptional recovery.

Based on this perspective, the present study aimed to explore the patterns of psychological and behavioral processes associated with ADL improvements that exceed prognostic predictions in stroke survivors, by drawing on the experiential knowledge of occupational therapists. The present study is grounded in a core principle of the PD approach—namely, learning from exceptional cases and translating those insights into strategies applicable to a broader population. The study findings may offer meaningful implications for promoting ADL improvement among stroke survivors and thereby contribute to informing rehabilitation practice.

## 2 Materials and methods

### 2.1 Definition of terms

In this study, stroke survivors whose total motor FIM score at discharge exceeded the predicted value by 10 points or more—based on the prediction formula developed by Tokunaga et al. (7)—were operationally defined as “patients who demonstrated PD exhibiting ADL improvement beyond prognostic expectations.” This threshold was selected because a deviation of more than 10 points on the motor FIM corresponds to a clinically meaningful shift in ADL independence level; a patient's level of ADL independence changes approximately every 10 points on this scale, with scores below 50 indicating complete assistance, 50–69 indicating incomplete assistance, 70 or above indicating self-care independence, and 80 or above indicating walking independence (10, 16). This prediction formula has been validated in a previous study, which reported an intraclass correlation coefficient of 0.84 between the actual and predicted values (10).

### 2.2 Participants

Participants were selected by purposeful sampling (17) from a rehabilitation hospital in Japan between October 2024 and January 2025. The rehabilitation system in Japan emphasizes intensive, goal-oriented therapy in inpatient settings, and this provides a rich context for exploring therapists’ perspectives on stroke recovery. The inclusion criteria were: 1) occupational therapists who had provided therapy to stroke survivors showing ADL improvements that exceeded prognostic predictions between April 2022 and March 2024, and 2) those with at least 1 year of experience working in rehabilitation wards. The only exclusion criterion was refusal to participate in the study. A total of 10 occupational therapists were identified as meeting the inclusion criteria. Consistent with the concurrent data collection and analysis approach, participants were recruited sequentially; the researcher (R.K.) invited therapists one at a time, with due consideration of diversity in gender, years of experience as an occupational therapist, and experience in specialized rehabilitation wards. Recruitment ceased after eight participants had been enrolled, as thematic saturation was reached prior to exhausting the full pool of eligible participants. R.K. is an external researcher affiliated with a different institution from the study site. While some participants had previously met the researcher through academic or clinical activities, others were not personally acquainted. The researcher had no supervisory or evaluative authority over any participant.

This study was approved by the ethics committees of Tokyo Metropolitan University (approval No. 24037). Written and verbal explanations of the study were provided to all participants, and written informed consent was obtained before interviews. This study adheres to the Standards for Reporting Qualitative Research (SRQR) (18).

### 2.3 Procedures

This study employed a qualitative descriptive design. Qualitative description is a methodological approach that prioritizes a comprehensive summary of phenomena as experienced and expressed by participants, without imposing a strong theoretical interpretive framework (19). This approach is consistent with the present study's aim to explore how occupational therapists perceive and interpret the psychological and behavioral processes of patients who demonstrated PD. Semi-structured interviews were conducted to allow participants to speak freely. Data were collected entirely through interviews, and the interview guide was developed based on the PD framework (11) and the MOHO (13), and was pre-tested by two occupational therapists not included in the main study before the study commenced, with minor revisions made based on their feedback.

Interviews with participants focused on how the patients who demonstrated PD coped with their post-stroke situation and how their ADLs improved. Furthermore, the interviews explored the participants’ perceptions of the factors that facilitated the patients’ recovery. Prior to each interview, participants were provided with a list of patients who met the criteria for PD, enabling them to identify specific patients they had treated. Participants were asked to recall their interactions with patients they had treated within the past 2 years. To support accurate recall, they were encouraged to refer to clinical records as needed. An overview of the interview guide is presented in Supplementary Table 1. To ensure that participants could respond efficiently, the interview guide was presented to them in advance of the interview sessions. Each interview was conducted individually and lasted approximately 50–60 min, as participants had been informed in advance. Where participants had worked with more than one patient meeting the PD criteria, the interview questions were applied sequentially to each patient; advance familiarity with the guide and interview format allowed this to be completed within the time that had been informed in advance. All interviews were audio-recorded, and detailed field notes that captured observations and contextual information were recorded. The shared professional background of R.K. and the participants as occupational therapists fostered a collegial conversational atmosphere from the outset. When participants had difficulty recalling specific clinical details, R.K. used open-ended prompts and encouraged reference to clinical records as needed, while avoiding leading questions or interpretations. In instances where communication became strained or unclear, R.K. acknowledged the participant's perspective, redirected the discussion to a related topic within the interview guide, and later returned to the original question to facilitate continued reflection and discussion. Following each interview, transcripts were returned to participants for review and correction prior to analysis. Interviews were continued with additional participants based on the principle of thematic saturation (20), defined as the point at which no new codes or subthemes emerged from subsequent interviews. Saturation was determined collaboratively by the research team following the seventh interview, with one additional interview conducted to confirm that no further themes emerged.

All interviews were conducted by R.K., an occupational therapist and researcher (PhD) with more than 13 years of experience in stroke rehabilitation and a research background in prognostic prediction. This clinical and academic expertise was considered to provide valuable insight into exploring the patterns of psychological and behavioral processes in patients who demonstrated PD. Nonetheless, it was acknowledged that his prior clinical experience could potentially introduce bias into data collection and analysis. To mitigate this, R.K. made a conscious effort to remain aware of his positionality and engaged in continuous reflexivity throughout both the interview and data analysis processes. The second author (N.K.) holds expertise in occupational therapy theory and qualitative research methodology, and both researchers have training in qualitative inquiry.

### 2.4 Analysis

Data were analyzed inductively using Reflexive Thematic Analysis (RTA) (21)—a method that identifies common themes and patterns from interviews and narratives. RTA is a flexible method that does not impose strict criteria on sample size, as it emphasizes the qualitative depth of data and the richness of interpretation. Following Braun and Clarke's framework (21), the analysis was conducted inductively in six phases: 1) familiarization with data, 2) generating initial codes, 3) searching for themes, 4) reviewing themes, 5) defining and naming themes, and 6) producing the report.

Data collection and analysis were conducted concurrently, with analysis commencing immediately after each participant's interview. To ensure rigor, R.K. engaged in continuous reflexivity throughout the analytical process, remaining attentive to how his clinical background and prior familiarity with the cohort might shape interpretation. Reflexive engagement was documented through analytical memos maintained throughout the analysis, in which R.K. recorded his interpretive decisions, emerging assumptions, and their potential influence on the developing analysis. In keeping with the reflexive nature of RTA, Phases 1–3—familiarization with data, generating initial codes, and searching for themes—were conducted solely by R.K., allowing for sustained and immersive engagement with the data. Phases 4–6 involved collaborative review and discussion with N.K., with any disagreements resolved through repeated discussion and independent third-party review where necessary. Additionally, we asked an independent third party who was not involved in this study to review the final codes and themes to assess their validity, thereby enhancing the credibility of the analysis. The validity of the analysis was enhanced through member checking with participants. NVivo 14 software (QSR International, Inc., Cambridge, MA, USA) was used for data analysis.

## 3 Results

### 3.1 Participant characteristics

After completing the interviews with the seventh participant, it was confirmed that the data had converged around existing themes, and data saturation was ultimately reached with the collection of the eighth participant's data. An overview of the research participants is presented in Table 1. The study included a total of eight participants (two males and six females), with a mean of 7.0 ± 5.1 years of experience in occupational therapy and stroke rehabilitation. Between April 2022 and March 2024, participants had been involved in the rehabilitation of one to three patients who demonstrated PD. All interviews were conducted face-to-face, with an average interview duration of 53.9 min. Across all participants, a total of 16 patients (nine males and seven females) who demonstrated PD were included as the basis for reflection during the interviews.

**Table 1:** Overview of the research participants.

ID	Sex	OT Experience (Years)	Stroke Intervention Experience (Years)	Interview time (Minutes:Seconds)	Number of patients who demonstrated PD^a^
1	Male	18	18	48:13	1 (Male 1)
2	Female	9	9	54:55	2 (Male 2)
3	Female	3	3	54:10	1 (Female 1)
4	Male	5	5	55:31	3 (Male 2, Female 1)
5	Female	6	6	55:37	2 (Male 1, Female 1)
6	Female	2	2	51:03	2 (Male 1, Female 1)
7	Female	9	9	52:01	3 (Male 2, Female 1)
8	Female	4	4	60:03	2 (Female 2)

a Number of patients who demonstrated positive deviance in the study cohort during the study period.

### 3.2 Patterns of psychological and behavioral processes in patients who demonstrated PD

**Figure 1:**
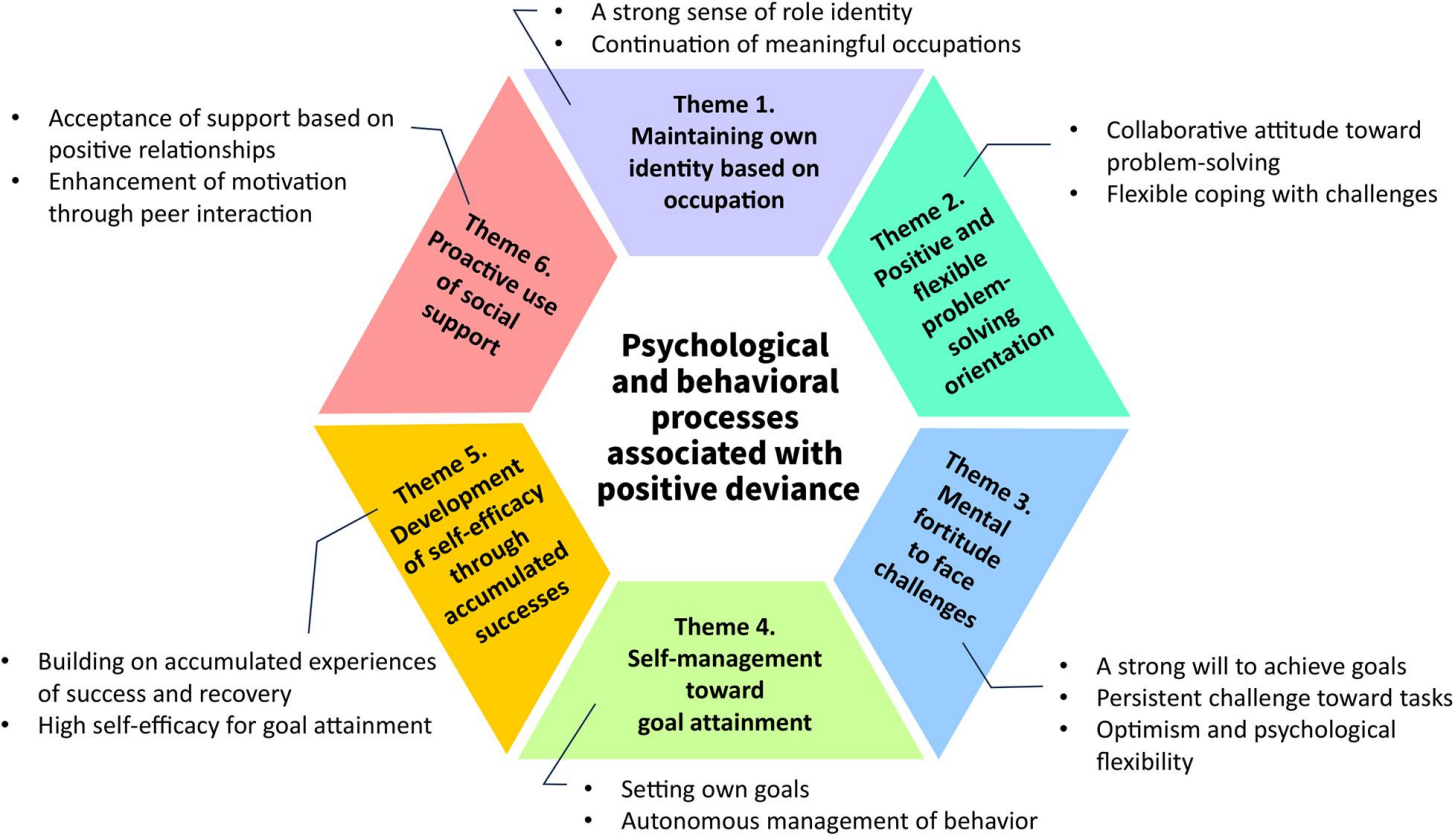
Psychological and behavioral processes associated with positive deviance in stroke rehabilitation as interpreted by occupational therapists: six themes and subthemes.

Through the RTA, this study generated themes reflecting how occupational therapists interpret the psychological and behavioral processes observed among patients who showed improvements in ADL beyond prognostic expectations. The analysis yielded 27 codes, which were organized into 13 subthemes and further integrated into six overarching themes: 1) maintaining own identity based on occupation, 2) positive and flexible problem-solving orientation, 3) mental fortitude to face challenges, 4) self-management toward goal attainment, 5) development of self-efficacy through accumulated successful experiences, and 6) proactive use of social support (Table 2). An overview of the themes and subthemes is presented in Figure 1. The following sections provide a detailed account of each theme.

**Table 2:** Themes, subthemes, and codes identified from the study

Themes	Subthemes	Codes
Maintaining own identity based on occupation	A strong sense of role identity	• Strong sense of responsibility for work, housework, and other roles
		• A sense of contribution to family and surroundings
	Continuation of meaningful occupations	• Have occupations that have been important since before the onset
		• Engage in meaningful occupations
Positive and flexible problem-solving orientation	Collaborative attitude toward problem-solving	• Receptive to, and acts on, advice with integrity
		• Understand and internalize the intended purpose and effects of the training
		• Consult others and seek advice about personal difficulties
	Flexible coping with challenges	• Recognize one's own abilities
		• Engage in trial and error to solve problems
		• Identify and select solutions that are appropriate for oneself
Mental fortitude to face challenges	A strong will to achieve goals	• Highly motivated to achieve their goals
		• Strong will and conviction
	Persistent challenge toward tasks	• Perseverance and effort in challenging circumstances
		• Take on challenges proactively without fear of failure
	Optimism and psychological flexibility	• Have a positive outlook on the future
		• Demonstrate psychological flexibility with optimism
Self-management toward goal attainment	Setting own goals	• Set clear and specific personal goals
	Autonomous management of behavior	• Engage in continuous self-directed practice
		• Engage in self-reliant actions
Development of self-efficacy through accumulated successful experiences	Building on accumulated experiences of success and recovery	• Recognize and value small progress positively
		• Recognize a sense of recovery
	High self-efficacy for goal attainment	• Demonstrate confidence in achieving goals
Proactive use of social support	Acceptance of support based on positive relationships	• Maintain positive relationships with others
		• Accept psychological support from others
		• Accept physical support from others
	Enhancement of motivation through peer interaction	• Engage actively with other patients
		• Incorporate influences from other patients

#### 3.2.1 Theme 1: maintaining own identity based on occupation

This theme comprises two subthemes: 1) a strong sense of role identity, and 2) continuation of meaningful occupations. Therapists perceived patients who demonstrated PD as showing an awareness of their own roles they had assumed prior to the onset of stroke—such as employment or domestic responsibilities—and showed a strong desire to continue engaging in meaningful occupations associated with those roles even after the onset. A notable process among these individuals was their proactive attitude toward continuing occupations that contributed to their identity, even during the early phase of hospitalization. One participant described a patient as follows:

It seemed that creating leather crafts as gifts for his grandchildren was a source of purpose in life for him. He had continued his leatherwork even while working as a company employee, and he mentioned that he had also given his creations to friends. He appeared to be truly passionate about it—during his hospital stay, he even made small items and gave them to the hospital staff. (Participant 1)

#### 3.2.2 Theme 2: positive and flexible problem-solving orientation

This theme comprises two subthemes: 1) collaborative attitude toward problem-solving, and 2) flexible coping with challenges. Therapists described patients who demonstrated PD as showing a willingness to accept advice and suggestions from others while also proactively expressing their difficulties when necessary. They actively engaged in exploring solutions in collaboration with their supporters, including occupational therapists. A participant described how the patient flexibly accepted various suggestions, as follows:

At mealtime, she had been using a spoon with her non-paralyzed left hand, but at one point, I suggested switching to using chopsticks with her paralyzed right hand. At that time, she was like, “I'll just give it a try,” you know? She wasn't negative about it at all. She was generally open to our suggestions. That included things like changes to her home environment—she was really flexible and willing to try almost anything. It made it easy for us to propose different options. (Participant 3)

Moreover, therapists interpreted these individuals as exhibiting adaptive coping strategies that reflected a positive and flexible problem-solving approach, engaging in repeated trial and error based on an awareness of their own capabilities and limitations, and ultimately taking the initiative to select strategies that best suited their needs.

He had thalamic pain in his upper and lower limbs, and it was like everything hurt when he bent them. But as the pain started to ease, he was the kind of person who could figure out how to cope by using his non-paralyzed side. He was always coming up with his own ways of doing things and trying stuff out on his own. (Participant 2)

#### 3.2.3 Theme 3: mental fortitude to face challenges

This theme comprises three subthemes: 1) a strong will to achieve goals, 2) persistent challenge toward tasks, and 3) optimism and psychological flexibility. Therapists perceived patients who demonstrated PD as showing a high level of motivation toward achieving their goals and as exhibiting persistent effort even under difficult circumstances. A participant described how the patient, despite physical limitations, was highly committed to rehabilitation in pursuit of their goals:

Yeah, there was definitely some effect from the paralysis. He ended up having shoulder pain, too. In the end, he was able to lift his arm a little, but there were still some limitations. His pinch strength was weak, but he somehow managed to thread the cords for the leatherwork, doing his best. His finger joints were stiff as well—they didn't move very smoothly or dexterously, but I think he just made do with what he had and did what he could. (Participant 1)

In addition, psychological flexibility—reflected in the ability to regulate negative emotions and maintain an optimistic and forward-looking perspective even when facing setbacks or failure—was commonly observed and interpreted by therapists. A participant described how the patient, after accepting the difficulty of returning to their previous lifestyle, shifted to a more positive and forward-looking attitude:

At first, I thought she might be the type to get a bit down, so I was a little worried about how things would go. But after a doctor told her that it would be difficult to return to how things were before, she said, “I've accepted it.” From then on, she seemed to shift to a more forward-looking mindset, like, “Okay, so what can I do from here?”. (Participant 5)

#### 3.2.4 Theme 4: self-management toward goal attainment

This theme comprises two subthemes: 1) setting own goals, and 2) autonomous management of behavior. Therapists described patients who demonstrated PD as tending to set realistic and clear goals and as consistently demonstrating self-directed engagement in rehabilitation to achieve them. One participant described how a patient proactively continued self-directed training from an early stage:

At first, her grip strength was weak—she couldn’t even open a plastic bottle cap. The paralysis was mild, but it seemed like her muscle strength had gone down. So, I suggested some self-training exercises to work on her grip strength, and she really stuck with them. After that, her grip got better pretty quickly. In that sense, I got the impression she was pretty proactive about doing self-training from early on in her hospitalization. (Participant 6)

In addition, patients who demonstrated PD were described as independently engaging in daily activities and regulated their behavior in a self-directed manner, without excessive reliance on healthcare providers.

He didn’t seem very dependent—he had this mindset of “I’ll take care of things myself” to some extent. So even without us prompting him, he would go ahead and do what he could on his own. That kind of proactive attitude really stood out to me. (Participant 5)

#### 3.2.5 Theme 5: development of self-efficacy through accumulated successful experiences

This theme comprises two subthemes: 1) building on accumulated experiences of success and recovery, and 2) high self-efficacy for goal attainment. Therapists perceived patients who demonstrated PD as tending to positively interpret small steps forward during the rehabilitation process, and as gradually developing a stronger sense of self-efficacy through the accumulation of such experiences. One participant described how the patient was aware of their own recovery and reflected on it introspectively:

There were moments when she reflected on her own progress, saying things like, “Maybe I got better because I did it this way.” We also tried to guide her a little, and I think it was a good process overall, with us being able to provide positive feedback at the right moments. (Participant 4)

Furthermore, based on these cumulative experiences, patients who demonstrated PD were perceived as developing solid confidence in achieving their goals and showing a willingness to take on more challenging tasks.

He participated in cooking training during his hospitalization and made curry. He was pretty confident about it — kind of like, “I got this,” you know?. (Participant 6)

#### 3.2.6 Theme 6: proactive use of social support

This theme comprises two subthemes: 1) acceptance of support based on positive relationships, and 2) enhancement of motivation through peer interaction. Therapists observed patients who demonstrated PD as actively accepting both psychological and physical support from those around them, including family members and friends, while maintaining constructive social relationships. One participant noted that the patient's ongoing communication with their spouse contributed to emotional stability and engagement in rehabilitation:

His wife was incredibly supportive and always cheering him on. I often saw him calling her in the evenings, and she'd be encouraging him over the phone—it was a regular thing. (Participant 2)

In addition, therapists described peer interaction as another important source of motivational enhancement. Participants described how patients were influenced by observing the rehabilitation efforts of others in similar conditions, which fostered a sense of mutual encouragement.

She said that when she saw a patient on the same ward who was working hard on gait training, she said, “That’s really amazing.” She once said, “When I see her working hard, it makes me want to work hard too.” Those interactions seemed to have a very positive impact on her. (Participant 4)

## 4 Discussion

This study aimed to qualitatively explore the patterns of psychological and behavioral processes associated with patients who exhibited ADL improvement that exceeded prognostic expectations, based on the clinical experiences of occupational therapists. The key patterns associated with patients who demonstrated PD, as identified through RTA, were as follows: 1) maintaining own identity based on occupation, 2) positive and flexible problem-solving orientation, 3) mental fortitude to face challenges, 4) self-management toward goal attainment, 5) development of self-efficacy through accumulated successful experiences, and 6) proactive use of social support. Overall, as interpreted by occupational therapists, these themes illustrate that individuals who exceeded recovery expectations demonstrated a strong sense of self, proactive engagement in problem solving, and the ability to adaptively mobilize internal and external resources to pursue meaningful goals. The findings of this study extend existing knowledge on the importance of psychological and behavioral processes in stroke rehabilitation, by identifying these processes specifically within the context of positive deviance—a framework that has received limited attention in qualitative rehabilitation research.

Regarding maintaining own identity based on occupation, this process can be interpreted as aligning with the concept of “occupational identity” as proposed in the MOHO. Occupational identity is defined as “a composite sense of who one is and wishes to become as an occupational being, generated from one's history of occupational participation” (13). A previous study involving older adults requiring long-term care has reported that perceptions related to occupational identity—such as deriving enjoyment in daily life and the desire to meet the expectations of others—were significantly associated with greater independence in ADL (22). This finding is consistent with the results of the present study. Indeed, patients who demonstrated PD were perceived as having a clear awareness of their own roles, actively seeking opportunities to engage in meaningful occupations, and taking initiative toward continued participation. These findings suggest that, even after stroke, maintaining their own identity based on occupation may enhance motivation for occupational engagement, promote physical activity, and ultimately contribute to improved outcomes in ADL.

Stroke survivors are required to reconstruct their daily lives while coping with physical and cognitive impairments. In this context, therapists interpreted patients who demonstrated PD as exhibiting a positive and flexible problem-solving orientation. Nezu et al. emphasized that effective problem-solving involves perceiving challenges not as threats but as opportunities and engaging proactively in resolution rather than avoidance (23). Such active coping strategies foster realistic goal setting and enhance motivation for rehabilitation, thereby contributing to improvements in ADL (24, 25). Furthermore, among older adults, the development of problem-solving skills is associated with enhanced occupational performance (26). Taken together, these findings indicate that a positive and flexible problem-solving orientation may serve as a key facilitator of ADL improvement in stroke rehabilitation.

The mental fortitude to face challenges, as identified in this study, was considered similar to the concept of “psychological resilience.” Patients with high psychological resilience regulate their stress responses appropriately, recover quickly from adversity, and exhibit a proactive attitude toward treatment, all of which are suggested to contribute to improved outcomes (27, 28). In contrast, stroke survivors who exhibit negative affectivity, such as pessimism or anxiety, have significantly lower levels of functional independence at 3 months post-onset as compared to those without such affectivity (29). These findings suggest that higher psychological resilience plays an important role in enhancing active participation in rehabilitation and motivation toward treatment, contributing to better outcomes; the results of the present study were consistent with these reported findings.

As interpreted by the therapists, patients who demonstrated PD exhibited sustained engagement in self-management toward goal attainment as one of the key processes identified in this study. Among patients with chronic diseases, those who actively engaged in self-management exhibited increased physical activity, more structured routines, and improvements in functional ability (30, 31). To achieve effective self-management, it is necessary for patients to take responsibility for their own actions and to independently set goals and develop action plans (32). Furthermore, post-stroke self-management is enhanced through educational interventions, the provision of individualized information, and support aimed at increasing confidence in self-management (33, 34). Indeed, rehabilitation programs that incorporate self-management components improved ADL in patients after a stroke (35, 36). These findings suggest that self-management can be facilitated through appropriate support from healthcare providers, and that the nature of such support may further promote patient recovery.

As interpreted by the therapists, patients who demonstrated PD developed self-efficacy through the accumulation of small successes. Self-efficacy was defined as the confidence to carry out a course of action that is necessary to accomplish desired goals (37), and it constitutes a psychological factor that influences the persistence of effort toward goals and perseverance in the face of challenges (12). Indeed, self-efficacy has been identified as a predictor of functional independence in stroke survivors (38), which highlights its value as a psychological resource during the recovery process. The complexity of the sequelae of strokes can easily lead to a feeling of helplessness and lack of control and, even under such circumstances, therapists interpreted patients who demonstrated PD as perceiving small achievements positively and accumulating these as successful experiences, whereby the accumulation of such successful experiences gradually strengthened their self-efficacy and may be associated with more proactive engagement in further recovery. The results of this study supported the importance of these processes as factors associated with favorable recovery outcomes.

One of the processes identified in patients who demonstrated PD was their proactive use of social support, which included assistance from family, friends, and peers. Social support plays a crucial role in the recovery process of patients, and acceptance of external support has been shown to enhance resilience in the face of adversity and to promote greater autonomy (39, 40). Indeed, patients who received higher levels of social support showed significantly greater improvements in ADL compared to those with limited support (41). Furthermore, peer support not only provides useful information and practical strategies for addressing daily challenges during rehabilitation, but also contributes to strengthening self-efficacy, facilitating improvements in ADL and alleviating symptoms of depression and anxiety (42). However, stroke rehabilitation is frequently prolonged, and patients are prone to experiencing negative emotions, such as loneliness and feelings of inferiority. These emotions may lead to withdrawal from social interactions with family and friends, and could result in reduced utilization of social support (43). Based on these findings, determining how patients perceive, accept, and use available social support could be important in the recovery process.

The credibility of these findings is further supported by a recent qualitative meta-synthesis of stroke survivors’ lived experiences (44), which identified fractured identity and self-reconstruction, family co-recovery, and pathways of hope through peer networks and resilience as central themes of stroke survivorship. Several of these themes converge with the facilitating processes identified in the present study—particularly Theme 1 (maintaining own identity based on occupation), Theme 3 (mental fortitude to face challenges), and Theme 6 (proactive use of social support)—suggesting that the findings reported here resonate with what patients themselves describe as meaningful in their recovery.

Taken together, the six themes identified in this study are best understood not as isolated characteristics but as an interrelated set of modifiable processes that collectively underpin exceptional recovery. At the core of this constellation lies a strong sense of occupational identity (Theme 1), which appeared to serve as a foundational motivational anchor: patients who, as interpreted by therapists, maintained a clear sense of who they were and what they valued were more likely to set personally meaningful goals and engage in self-directed practice (Theme 4). This self-management, in turn, created repeated opportunities for small successes, which progressively strengthened self-efficacy (Theme 5). A positive and flexible problem-solving orientation (Theme 2) enabled patients to approach the setbacks of stroke recovery as challenges to be navigated, rather than avoided, a process further sustained by the mental fortitude and psychological flexibility captured in Theme 3. Finally, proactive use of social support (Theme 6) provided a relational scaffolding that reinforced all of the aforementioned processes; encouragement from family, peers, and therapists helped patients maintain their sense of identity, persist through difficulties, and sustain confidence in their recovery. Exceptional recovery may therefore depend not on any single factor but on the dynamic interplay among these processes, with each reinforcing the others over the course of rehabilitation.

These findings have important implications for clinical practice. Rather than targeting individual factors in isolation, therapists may facilitate recovery by creating conditions that enable patients to explore, reflect on, and act according to their own values and goals. For example, incorporating meaningful pre-stroke occupations into early assessment may simultaneously strengthen occupational identity, support goal setting, and provide opportunities for early success experiences. Importantly, the facilitating processes identified in this study do not appear to be unique to exceptional individuals, but may represent modifiable dimensions of rehabilitation practice that can be fostered in a broader population of stroke survivors. Accordingly, insights derived from patients who demonstrated PD may help inform therapeutic approaches for stroke rehabilitation more generally. However, these findings were derived from occupational therapists’ reflections on patients treated within a single rehabilitation hospital in Japan, and should therefore be interpreted within the specific regional and institutional context in which the study was conducted. Japanese inpatient stroke rehabilitation is characterized by intensive, goal-oriented therapy delivered within a relatively structured healthcare system, and these contextual features may have influenced how the identified psychological and behavioral processes were expressed and supported. Accordingly, transferability to other rehabilitation settings, healthcare systems, or cultural contexts should be considered with appropriate caution.

This study had some limitations. First, as this is a qualitative study, the findings may inevitably be influenced by the researchers’ own positions, experiences, and knowledge. However, the authors sought to minimize potential bias through rigorous data collection and member checking of the analysis results by participants. Second, the participants were limited to occupational therapists working in the metropolitan area, which may have constrained the diversity of perspectives and resulted in a partial understanding of the patterns of psychological and behavioral processes in patients who demonstrated PD. To achieve a more comprehensive understanding, future studies should incorporate perspectives from stroke survivors, their family members, and other healthcare professionals across diverse communities. In particular, interviews with and observations of stroke survivors who demonstrated PD are expected to provide direct insight into recovery processes that cannot be fully captured through therapist reports alone. In addition, including perspectives on patients who demonstrate negative deviance would provide complementary evidence and strengthen the credibility of the identified themes. Integrating these multifaceted perspectives is expected to enhance the validity of the findings and their applicability to clinical practice. Third, the interrelationships among the identified processes were not empirically examined. Therefore, future research should explore the connections and interactions between these processes to facilitate a more comprehensive understanding. Fourth, the findings may have been influenced by recall bias, as participants retrospectively reported their clinical experiences. Although they were encouraged to refer to clinical records to support accurate recall, this limitation cannot be entirely excluded. Future studies using real-time data collection would help to address this issue. Fifth, detailed demographic data on patients who demonstrated PD (e.g., age, employment status, and education level) were not available to the research team, as these data were stored in the database of the collaborating institution and were inaccessible due to privacy protection protocols. Future studies should aim to collect comprehensive demographic data to enable a more nuanced interpretation of the findings. Sixth, the possibility that sex-related differences may have influenced the psychological and behavioral processes associated with PD cannot be excluded. Future research should investigate whether these processes differ between males and females after stroke, and whether sex-specific approaches to rehabilitation may be warranted. Taken together, addressing these limitations in future studies will further strengthen the evidence base needed to translate the insights derived from positive deviance into actionable, individualized rehabilitation practice.

## 5 Conclusion

We explored the patterns of psychological and behavioral processes in stroke survivors who demonstrated post-stroke ADL improvements that exceeded prognostic predictions, based on the clinical experiences of occupational therapists. Participants interpreted patients who demonstrated PD as exhibiting the following psychological and behavioral processes: 1) maintaining their own identity based on occupation; 2) positive and flexible problem-solving orientation; 3) mental fortitude to face challenges; 4) self-management toward goal attainment; 5) development of self-efficacy through accumulated successful experiences; and 6) proactive use of social support. In stroke rehabilitation, these findings not only support the aim of functional improvement but also underscore the importance of interventions that focus on psychological and behavioral processes that emerge within rehabilitation contexts. Taken together, the identified processes suggest that exceptional recovery may not be predetermined by neurological or demographic factors alone, but may be cultivated through skilled, person-centered practice that attends to patients’ values, identity, and relational resources. These insights were generated within the specific context of intensive inpatient rehabilitation in Japan, and their applicability to other settings and populations should be explored in future research. Future research should aim to complement and further develop these insights through data collection from more diverse and multidimensional perspectives.

## Data Availability

The original contributions presented in the study are included in the article/Supplementary Material, further inquiries can be directed to the corresponding author.
