# Supplementary material for: Psychological and behavioral processes in stroke survivors with recovery exceeding prognostic expectations: a qualitative study of positive deviance
Source: Front Rehabil Sci. 2026 Jun 29;7:1743882. doi: 10.3389/fresc.2026.1743882 (PMC13357529; doi:10.3389/fresc.2026.1743882)
Supplement: Supplementary file 1 [file Table1.docx]

**Supplementary table 1**. Interview guide

| 1. What was the patient’s condition at the time of admission? |
| --- |
| 1. What was the patient's clinical course during hospitalization? |
| 1. How did the patient appear when engaging in daily rehabilitation sessions? |
| 1. What kind of actions did the patient take in order to achieve their goals? |
| 1. Which patient characteristics do you think contributed to their recovery? |
| 1. Were there any notable aspects of the patient’s family or social environment?   If so, what were they? |
| 1. Compared to other patients, what strengths of this patient stood out to you?   Please provide specific examples. |
| 1. Compared to other patients, what distinctive features or observable behaviors did this patient exhibit? |
| 1. Based on your rehabilitation experience with this patient, how do you think the insights gained can be applied in future practice? |
